# Supplementary figures and images for: Tissue culture and Agrobacterium-mediated genetic transformation of the oil crop sunflower
Source: PLoS One. 2024 May 9;19(5):e0298299. doi: 10.1371/journal.pone.0298299 (PMC11081250; doi:10.1371/journal.pone.0298299)

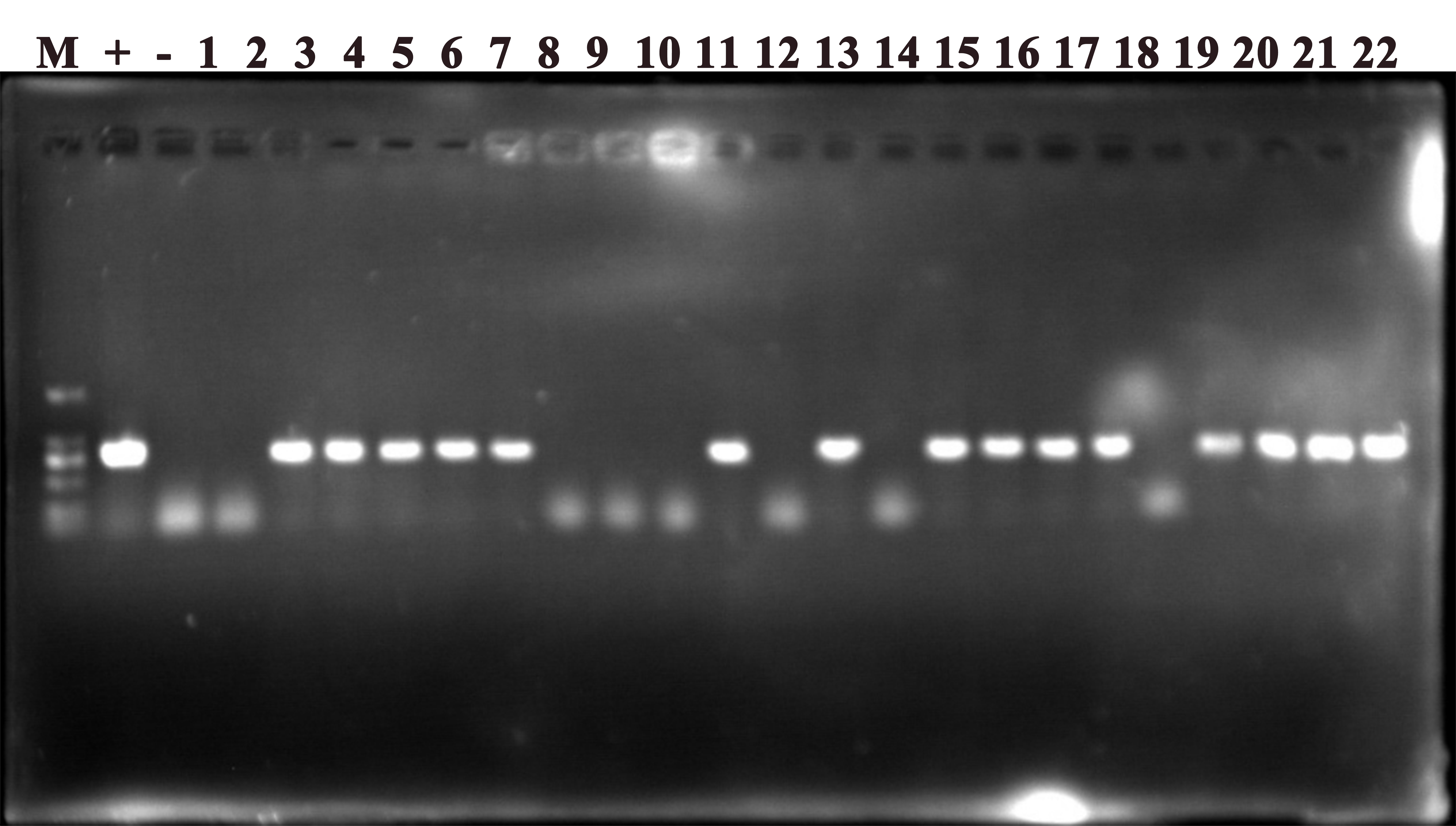

Supplement: S1 Fig — (JPG) [file pone.0298299.s001.jpg]

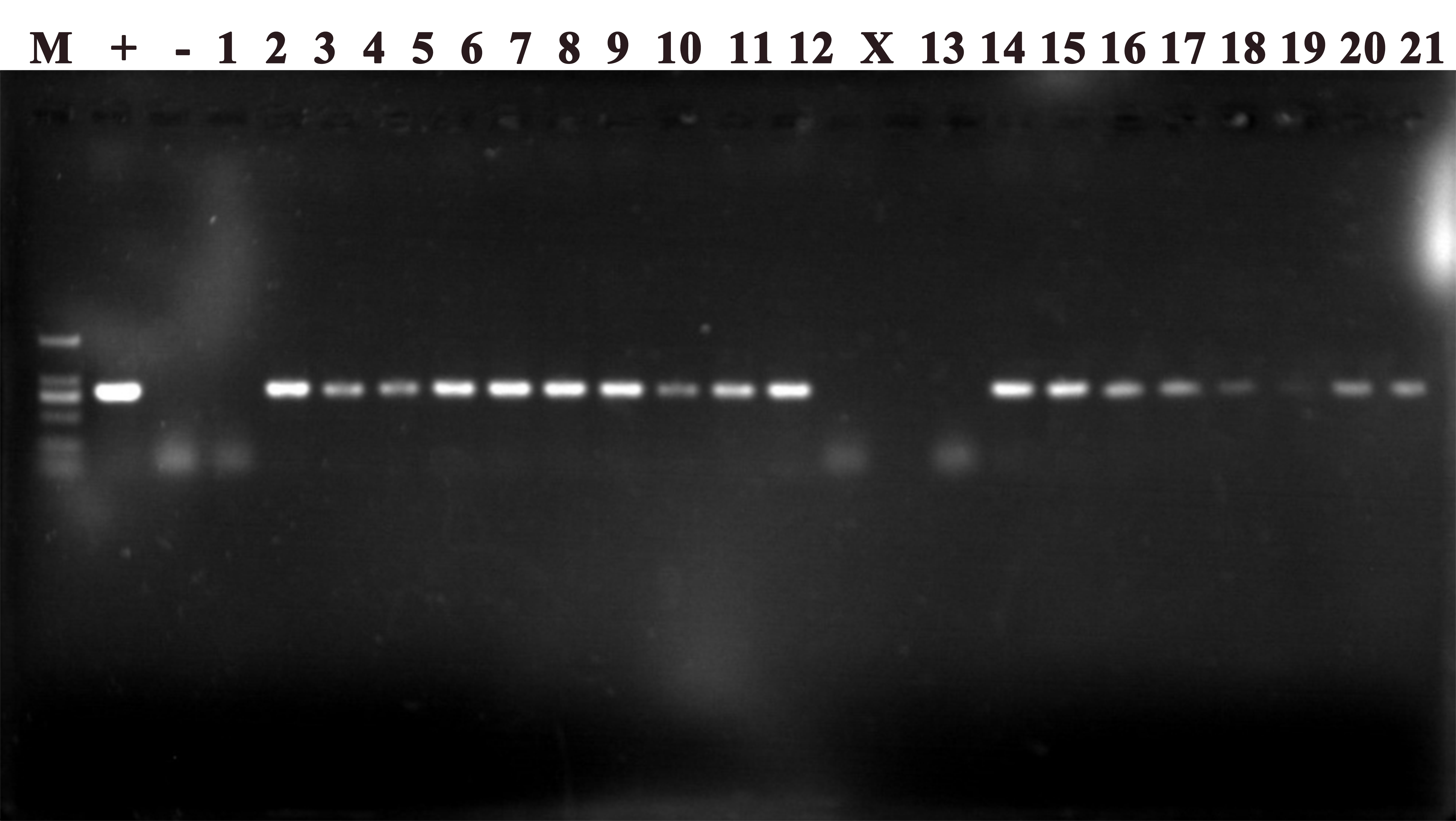

Supplement: S2 Fig — (JPG) [file pone.0298299.s002.jpg]
