# Supplementary material for: Tissue culture and Agrobacterium-mediated genetic transformation of the oil crop sunflower
Source: PLoS One. 2024 May 9;19(5):e0298299. doi: 10.1371/journal.pone.0298299 (PMC11081250; doi:10.1371/journal.pone.0298299)
Supplement: S1 Table — (DOCX) [file pone.0298299.s003.docx]

**Supplementary table 1** Different hormone combinations induce adventitious shoots directly through three types of explants

| Treatment group | Plant growth regulator /mg·L^-1^ Explants response /% |
| --- | --- |
|  | IAA 6-BA KT Cotyledon Hypocotyl Leaf |
| 1 0.01 0.0 2.0 0.0^b^ 0.0^c^ 0.0^b^  2 0.01 0.0 4.0 0.0^b^  25.0±1.0^a^ 0.0^b^  3 0.01 0.0 6.0 0.0^b^ 0.0^c^ 0.0^b^  4 0.01 0.0 8.0 0.0^b^ 0.0^c^ 0.0^b^  5 0.01 0.0 10.0 0.0^b^ 0.0^c^ 0.0^b^  6 0.01 0.5 0.0 0.0^b^ 0.0^c^ 0.0^b^  7 0.01 0.9 0.0 0.0^b^ 0.0^c^ 12.5±0.6^a^  8 0.01 1.2 0.0 0.0^b^ 0.0^c^ 12.5±0.0^a^  9 0.01 1.5 0.0 0.0^b^ 0.0^c^ 0.0^b^  10 0.01 2.5 0.0 0.0^b^ 0.0^c^ 0.0^b^  11 0.01 3.0 0.0 0.0^b^ 0.0^c^ 0.0^b^  12 0.01 5.0 0.0 16.7±0.6^a^ 33.3±0.6^b^ 0.0^b^  13  0.01 7.0 0.0 0.0^b^ 0.0^c^ 0.0^b^  14 0.01 9.0 0.0 0.0^b^ 0.0^c^ 0.0^b^  15 0.03 2.5 0.0 0.0^b^ 0.0^c^ 12.5±0.6^a^  16 0.05 0.0 2.0 0.0^b^ 0.0^c^ 0.0^b^  17 0.05 0.0 4.0 0.0^b^ 13.0±1.0^b^ 0.0^b^  18 0.05 0.0 6.0 0.0^b^ 0.0^c^ 0.0^b^  19 0.05 0.0 8.0 0.0^b^ 0.0^c^ 0.0^b^  20 0.05 0.0 10.0 0.0^b^ 0.0^c^ 0.0^b^  21 0.05 0.5 0.0 0.0^b^ 0.0^c^ 0.0^b^  22 0.05 0.9 0.0 0.0^b^ 0.0^c^ 12.5±0.0^a^  23 0.05 1.2 0.0 0.0^b^ 0.0^c^ 12.5±1.0^a^  24 0.05 1.5 0.0 0.0^b^ 0.0^c^ 12.5±0.6^a^  25 0.05 2.5 0.0 0.0^b^ 0.0^c^ 0.0^b^  26 0.05 3.0 0.0 0.0^b^ 12.5±0.6^b^ 0.0^b^  27 0.05 5.0 0.0 0.0^b^ 50±0.6^a^ 0.0^b^  28 0.05 7.0 0.0 0.0^b^ 0.0^c^ 0.0^b^  29 0.05 9.0 0.0 0.0^b^ 0.0^c^ 0.0^b^  30 0.1 0.0 2.0 0.0^b^ 0.0^c^ 0.0^b^  31 0.1 0.0 4.0 0.0^b^ 0.0^c^ 0.0^b^  32 0.1 0.0 6.0 0.0^b^ 0.0^c^ 0.0^b^  33 0.1 0.0 8.0 0.0^b^ 0.0^c^ 0.0^b^  34 0.1 0.0 10.0 0.0^b^ 0.0^c^ 0.0^b^  35 0.1 0.5 0.0 0.0^b^ 0.0^c^ 0.0^b^  36 0.1 0.9 0.0 0.0^b^ 0.0^c^ 12.5±0.0^a^  37 0.1 1.2 0.0 0.0^b^ 0.0^c^ 0.0^b^  38 0.1 1.5 0.0 0.0^b^ 0.0^c^ 0.0^b^ 39 0.1 3.0 0.0 0.0^b^ 0.0^c^ 0.0^b^  40 0.1 5.0 0.0 0.0^b^ 0.0^c^ 0.0^b^  41 0.1 7.0 0.0 0.0^b^ 0.0^c^ 0.0^b^  42 0.1 9.0 0.0 0.0^b^ 0.0^c^ 0.0^b^ | |

**Notes:** Experimental data were analysed using one-way variance (ANOVA). Differences in treatment means were expressed as the mean ± standard error (SE) of the experiments, and Tukey's Least Significant Difference (LSD) test was applied to detect significant differences at p<0.05 for comparison.
