# Supplementary material for: Tissue culture and Agrobacterium-mediated genetic transformation of the oil crop sunflower
Source: PLoS One. 2024 May 9;19(5):e0298299. doi: 10.1371/journal.pone.0298299 (PMC11081250; doi:10.1371/journal.pone.0298299)
Supplement: S2 Table — (DOCX) [file pone.0298299.s004.docx]

**Supplementary table 2** Different hormone combinations induce adventitious shoots directly through cotyledonary

| Treatment group | Plant growth regulator /mg·L^-1^ Explants response /% The mean ± (SE) |
| --- | --- |
|  | IAA 6-BA KT Cotyledonary nodes |
| 1 0.05 0.25 0.0 100.0  83.3 83.3 88.7±5.67^ab^  2 0.05 0.3 0.0 83.3 66.7 83.3 77.8±5.56^ab^  3 0.05 0.35 0.0 83.3 83.3 83.3 83.3±0^ab^  4 0.05 1.0 0.0 66.7 83.3 83.3 77.8±5.56^ab^  5 0.05 3.0 0.0 66.7 66.7 83.3 72.2±5.56^abc^  6 0.1 0.5 0.0 50.0 66.7 66.7 61.1±5.56^abc^  7 0.1 0.6 0.0 83.3 66.7 66.7 72.2±5.56^abc^  8 0.1 0.7 0.0 83.3 83.3 66.7 77.8±5.56^ab^  9 0.1 1.0 0.0 50.0 66.7 66.7 55.6±5.56^bcd^  10 0.1 3.0 0.0 33.3 33.3 0.0 22.2±11.11^d^  11 0.05 0.0 0.5 100.0 83.3 83.3 88.9±5.56^ab^  12 0.05 0.0 1.0 83.3 100.0 66.7 83.3±9.62^ab^  13  0.05 0.0 2.0 83.3 100.0 100.0 94.4±5.56^a^  14 0.05 0.0 4.0 83.3 50.0 66.7 66.7±9.62^abc^  15 0.05 0.0 6.0 83.3 66.7 66.7 72.2±5.56^abc^  16 0.1 0.0 0.5 100.0 66.7 83.3 83.3±9.62^ab^  17 0.1 0.0 1.0 66.7 83.3 83.3 77.8±5.56^ab^  18 0.1 0.0 2.0 66.7 66.7 83.3 72.2±5.56^abc^  19 0.1 0.0 4.0 50.0 66.7 50.0 55.6±5.56^bcd^  20 0.1 0.0 6.0 33.3 33.3 50.0 38.9±2.53^cd^ | |

**Notes:** Experimental data were analysed using one-way variance (ANOVA). Differences in treatment means were expressed as the mean ± standard error (SE) of the experiments, and Tukey's Least Significant Difference (LSD) test was applied to detect significant differences at p<0.05 for comparison.
