# Supplementary material for: Tissue culture and Agrobacterium-mediated genetic transformation of the oil crop sunflower
Source: PLoS One. 2024 May 9;19(5):e0298299. doi: 10.1371/journal.pone.0298299 (PMC11081250; doi:10.1371/journal.pone.0298299)
Supplement: Attachment — Submitted filename: Response to Reviewers.docx [file pone.0298299.s005.docx]

Dear academic editor Vijay Kumar ,

Thank you very much for your careful reading of this thesis and for your valuable comments. I have revised the content of the thesis in the light of the following issues.

1. Please ensure that your manuscript meets PLOS ONE's style requirements, including those for file naming. The PLOS ONE style templates can be found at
   https://journals.plos.org/plosone/s/file?id=wjVg/PLOSOne_formatting_sample_main_body.pdf and
   <https://journals.plos.org/plosone/s/file?id=ba62/PLOSOne_formatting_sample_title_authors_affiliations.pdf>

**I have reworked the article according to PLOS ONE's journal style formatting requirements.**

2. We note that the grant information you provided in the ‘Funding Information’ and ‘Financial Disclosure’ sections do not match.

When you resubmit, please ensure that you provide the correct grant numbers for the awards you received for your study in the ‘Funding Information’ section.

**I have re-checked and re-determined this section and made changes.**

3. Thank you for stating the following financial disclosure: 
 " This work was financially supported by the Natural Science Foundation of Xinjiang Uygur Autonomous Region (2020D01C020), Project 4 of Xinjiang Autonomous Region Major Special Project (2022A03004-4) and the Outstanding Doctoral Researchers Student Research Innovation Project in Xinjiang University (XJU2022BS050). They provided adequate funding for this study."

Please state what role the funders took in the study.  If the funders had no role, please state: ""The funders had no role in study design, data collection and analysis, decision to publish, or preparation of the manuscript."" 
If this statement is not correct you must amend it as needed. 
Please include this amended Role of Funder statement in your cover letter; we will change the online submission form on your behalf.

**This work was financially supported by the Natural Science Foundation of Xinjiang Uygur Autonomous Region (2020D01C020), Project 4 of Xinjiang Autonomous Region Major Special Project (2022A03004-4) and the Outstanding Doctoral Researchers Student Research Innovation Project in Xinjiang University (XJU2022BS050). The funders had no role in study design, data collection and analysis, decision to publish, or preparation of the manuscript.**

**The funder role statement changes have been added to the cover letter.**

4. Your ethics statement should only appear in the Methods section of your manuscript. If your ethics statement is written in any section besides the Methods, please move it to the Methods section and delete it from any other section. Please ensure that your ethics statement is included in your manuscript, as the ethics statement entered into the online submission form will not be published alongside your manuscript.

**My ethics statement has been moved to the methods section and removed from any other section.**

5. Please include a separate caption for each figure in your manuscript.

**I have added a separate caption for each figure in my manuscript.**

6. Please include captions for your Supporting Information files at the end of your manuscript, and update any in-text citations to match accordingly. Please see our Supporting Information guidelines for more information: <http://journals.plos.org/plosone/s/supporting-information.>

**I have added the support information at the end of the article. The additional data for this section is stored in the online site Figshare.**

7. PLOS ONE now requires that authors provide the original uncropped and unadjusted images underlying all blot or gel results reported in a submission’s figures or Supporting Information files. This policy and the journal’s other requirements for blot/gel reporting and figure preparation are described in detail at https://journals.plos.org/plosone/s/figures#loc-blot-and-gel-reporting-requirements and https://journals.plos.org/plosone/s/figures#loc-preparing-figures-from-image-files. When you submit your revised manuscript, please ensure that your figures adhere fully to these guidelines and provide the original underlying images for all blot or gel data reported in your submission. See the following link for instructions on providing the original image data: https://journals.plos.org/plosone/s/figures#loc-original-images-for-blots-and-gels.
    **I have submitted the original uncropped and unadjusted images of the gel to the online site figshare as required by the journal and mentioned in the cocverletter.**

5. Review Comments to the Author

Please use the space provided to explain your answers to the questions above. You may also include additional comments for the author, including concerns about dual publication, research ethics, or publication ethics. (Please upload your review as an attachment if it exceeds 20,000 characters)

Reviewer #1: Gene transformation is a pivotal procedure for gene editing breeding. This paper use a dominate cultivar to establish the approach, which is usually a very challenging process. This work make the cultivar amenable to gene editing. This is very good.

There is no big problems in this paper. I would prefer an sound explanation why the blue color is evenly appeared on the whole tissue after GUS staining.

**Thank you for your question. This is because the vector pBI121 used on our transgene contains a 35S strong constitutive promoter. Changing the promoter enables the *GUS* gene to initiate expression in all tissues.**

There also some minor writing formats errors. Please see below:

Some sentence are incomplete: "Next, were transferred to resistance screening“
Some braces lack space between the previous words: "medium(MS"
Some spaces also missed between two word.
Please make efforts on these writing errors.

**These errors were created due to our negligence and we have corrected them accordingly.**

Reviewer #2: The manuscript entitled “Tissue Culture and Agrobacterium-mediated Genetic Transformation of the Oil Crop Sunflower” studied regeneration and genetic transformation optimization in sunflower. The manuscript can be accepted after major revisions.

In the abstract section, “In this study, we selected different explants to establish a regeneration system and obtained that direct induction of adventitious buds and rooting by cotyledon nodes was in MS more suitable for the combination of 0.05 mg/L IAA 2.0 mg/L KT, and add 0.9 mg/L IBA.” This sentence needs to be rephrased for better understanding.
In the abstract section, “The rate of resistant seedlings as the highest transformation efficiency reached 8.0 % under the condition of 45 mg/L kanamycin, a bacterial density of OD600 0.8, infection time of 30 min and 3 days of co-cultivation” this sentence needs to change authors should mention optimization parameters in first then need to conclude the transformation efficiency.
 **Thank you for your detailed questions that have made this article better. We have revised them one by one.**

The authors mentioned they used 150.0 mg/L Acetosyringone in transformation studies, which is very high; it should be in μM.
 **These errors were created due to our negligence and we have corrected them accordingly.**

In Figure 4. Flow diagram of the tissue culture and regeneration for the ZADT. In the place of the Germination of sterile seeds image, the authors need to include the seedling image.

**This image has been modified as per the suggestions you have given.**

Figure 6. Expression of a GUS reporter gene from the 35S promoter in resistant seedlings of ZADT. GUS staining and PCR amplification images are poor; they must be replaced with good images.

**We were fortunate to find genomes of plant material extracted from previous experiments. The images have been re-replaced.**

Figure 8. Detection of the GUS gene of ZADT-positive plants grown for 2 months gel image is very poor, and a ladder needs to be added.

**We were fortunate to find genomes of plant material extracted from previous experiments. The images have been re-replaced.**

The author must clearly mention the selectable marker gene (nptII) name in the Genetic Transformation of Cotyledonary Nodes section.
 **Based on your suggestions, we have made changes in the corresponding places.**

Check the spelling of ‘convinient’ throughout the manuscript.
Check the spelling of ‘these system’ in the conclusion section.
Check the spelling of ‘flflower soil’ in the Regeneration optimization section.
The English language of the manuscript is very poor, and there are many grammatical mistakes that need to be corrected.

**Thanks to your suggestion, we have re-corrected all of this article for word and sentence grammar.**

The authors calculated the transformation efficiency by GUS staining or PCR amplification.? please clarify.

**We calculated the transformation efficiency by PCR amplification. This part we have revised and mentioned at the right place in the article.**

Authors need to mention keywords in the manuscript, and it should be in alphabetical order.

**Thanks for the reminder that the keywords have been added at the bottom of the abstract.**

Authors need to cite the latest articles instead of citing old articles.

**Regarding citations, we have attention to new and relevant literature reports. It is estimated that it is still incomplete, and we have added the latest reported literature in this revision. However, regarding sunflower-related reports, there are fewer recent studies, mainly some earlier reports.**

Reviewer #3: Observation: Please split it into two parts for a better understanding of the sentence. “Plant regeneration protocol is usually a prerequisite to the genetic transformation, the regeneration conditions are determined by optimising factors such as genotype, explant type and concentration of the inducing hormones.”
Observation: Too lengthy sentence. Please split it. “Usually, genotype
specificity influences the regeneration of most plants, such as cotton[7], sunflower[6],
etc., so it is essential to develop a reliable and reproducible regeneration protocol for
different genotypes or varieties; mullein(Casuarina equisetifolia) stem segments can
induce of callus, adventitious buds and adventitious roots through different hormone
combinations[8], as do garland chrysanthemum[9].
 Observation: Delete this part. It is not required. “plant growth regulators (PGRs) are synthetic chemicals that can replace its functions in vitro and are essential for the plant tissue culture process.”
 Observation: Delete or rewrite this paragraph. “Among them, Agrobacterium-mediated method is the most practical and inexpensive technique for the most plants. The GUS encoding
β-glucuronidase[27] is often as a reporter gene to detect transformation efficiency by
histochemical staining and sensitive fluorescence quantification of enzyme activity.”

**Thank you for your detailed questions that have made this article better. We have revised them one by one.**
